# Between the High Ideals and Reality: Managing COVID-19 Vaccine Nationalism

**DOI:** 10.1017/err.2021.9

**Published:** 2021-03-19

**Authors:** Lukasz GRUSZCZYNSKI, Chien-huei WU

**Affiliations:** * Associate Professor, Kozminski University, Warsaw, Poland; Research Fellow, Centre for Social Sciences – Institute for Legal Studies, Budapest, Hungary; email: lgruszczynski@kozminski.edu.pl.; ** Associate Research Professor, Institute of European and American Studies, Academia Sinica, Taipei, Taiwan; email: wch@sinica.edu.tw.

## Abstract

This report examines what has come to be known as “vaccine nationalism” through the lens of the early experience with the COVID-19 vaccination process. After explaining the meaning of the term, this report investigates how this phenomenon has manifested during the COVID-19 pandemic, identifying its epidemiological, economical, ethical and legal aspects. It also looks at the different international initiatives that have been adopted to deal with it, concentrating in this context on the COVAX project. The report concludes that the success of these initiatives has been limited. It also observes that COVID-19 vaccine nationalism appears to be a phenomenon that is characteristic of the high-income Western countries, while in aspiring non-Western powers the vaccine crisis is primarily seen as a way to advance their geopolitical goals.

## I. Introduction

The COVID-19 pandemic is one of the biggest challenges faced by humanity in the last several decades. So far, 119 million cases have been identified and almost 2.64 million people have died.^1^ However, due to limited testing capacity, in reality both numbers are much higher. For example, the Russian Deputy Prime Minister, Tatiana Golikova, has recently admitted that approximately three times more Russians have died due to coronavirus than officially reported.^2^ Many other countries face a similar – if not larger – problem. This public health emergency has also been accompanied by an economic crisis, with the world witnessing in 2020 the biggest contraction of the aggregated gross domestic product (GDP) since World War II. While various economic models predict an economic recovery in 2021, most likely it will be weaker than initially anticipated.

It is therefore not surprising that everyone has been waiting for the commencement of the vaccination process. Unfortunately, it has quickly turned out that the supply of vaccines is limited and mainly directed to rich regions of the world, with many developing countries left behind. The much-awaited remedy has created its own problems.

The objective of this report is to examine what has come to be known as “vaccine nationalism” through the lens of the early experience with the COVID-19 vaccination process. To this end, this report proceeds as follows: Section II explains the meaning of the term, and Section III discusses how this phenomenon has manifested during the COVID-19 pandemic, identifying its epidemiological, economical, ethical and legal aspects. Section IV looks at the different initiatives – with the COVID-19 Vaccine Global Access Facility (COVAX) currently being the most ambitious one – that have been adopted to address the problems created by vaccine nationalism. Section V offers brief conclusions.

## II. What is vaccine nationalism?

Vaccine nationalism is a buzzword coined by public health experts to describe a situation whereby governments take unilateral actions to provide their own populations with access to vaccines ahead of other countries.^3^ While governments can take different steps to achieve this objective, the most common approach is to secure supplies through contracts with pharmaceutical manufacturers that prioritise their orders either because they are willing to offer a higher price or because they contract for the supplies at the pre-approval stage, thus taking a part of the risks connected with the development of a new vaccine. Since production capacity is always limited when a vaccine is launched, after their demand is satisfied, very little – if anything – is left for other states. The short-term gains of such a strategy are clear. First, it can help governments politically by pleasing an electorate frustrated with prolonged economic and social disruptions (the now-famous “return to normal”). This in turn may boost the level of trust in governments and enhance their approval ratings. Second, such a strategy also makes it possible to reopen national economies faster. Governments can ease existing sanitary restrictions and in doing so support the recovery and reduce the burden on public finances. Third, vaccines can also be used strategically as a part of a country’s diplomatic repertoire. Of course, vaccine nationalism also comes with its costs – an aspect to which we return in Section III.

While the term “vaccine nationalism” has become widely used in the context of the COVID-19 pandemic,^4^ this type of behaviour is by no means new. Probably the most well-known example from the past concerns access to drugs against HIV. Highly active antiretroviral therapy (HAART), based on a combination of various antiviral drugs, was introduced in 1996. The therapy proved to be very effective and resulted in a sharp decrease in mortality rates among infected persons and significant improvements in the quality of life of persons with AIDS. However, because of the demand from developed countries and the high price of the therapy, for years its availability in the developing parts of the world remained very limited. The developed states, once they satisfied the demands of their domestic markets, were apparently more interested in securing profits for their pharmaceutical companies than in guaranteeing broad access to the life-saving drugs.^5^ More recently, a similar situation occurred with respect to the H1N1 vaccines. Some developed countries with vaccine production facilities introduced export restrictions (or threatened to do so), while others placed pre-purchase orders, reserving most of the vaccines that were capable of being manufactured at that time. Consequently, poorer nations were left behind, with no or very limited access to the vaccines.^6^ Their availability only improved when the H1N1 pandemic started to decline.

## III. Vaccine nationalism during the COVID-19 pandemic

So how has vaccine nationalism manifested itself during the current pandemic? Its early signs were already visible in March 2020 when the USA tried to take over the German company CureVac, or at least to ensure that it had exclusive access to the potential vaccine once it was developed.^7^ Such a move fitted very well into Trump’s vision of international affairs, characterised “by endemic conflict, relative gains, insecurity and asymmetry, [and] self-sufficiency – rather than … cooperation”,^8^ and it corresponded with the general transactional approach to global governance taken by the Trump administration. But other countries have not acted very differently. As the pandemic progressed, several developed states – including not only the USA but also the UK, the European Union (EU) acting on behalf of its Member States, Canada and Japan – concluded a number of bilateral agreements with pharmaceutical companies to secure their supplies of the future vaccines. Consequently, by mid-January 2021, these countries locked up about 60% of the 7 billion vaccines that were sold, despite the fact that they represented only 14% of the global population.^9^ For example, Canada has ordered five times more vaccines than its entire population. All of this means that at least 90% of people in lower-income countries will not be vaccinated by the end of 2021.^10^ The high-income countries have also been unanimous in blocking the work on the temporary waiver of certain obligations of the Agreement on Trade Related Aspects of Intellectual Property Rights (TRIPS).^11^ The proposal was made in October 2020 by India and South Africa and sought to make it easier for developing countries to produce COVID-19 vaccines and related drugs.^12^ The USA, the UK and the EU have argued – unpersuasively in our opinion – that such a move would restrain innovation by discouraging the pharmaceutical companies for further investment in the research and development of SARS-CoV-2 vaccines.

The asymmetries among the countries are quite visible when one looks at the current vaccination rates across the globe. As of 9 March 2021, the top of the list was dominated by high-income states, with Israel being the leader (103.71%), followed by the United Arab Emirates (63.95%), the UK (35.45%) and the USA (28.31%). Although the current figure for the EU as whole is a moderate 9.94%, the rate for Africa stands at the level of 0.41%, for Asia it stands at 2.54% and for South America it stands at 4.53%.^13^ Many of the lowest-income countries have not yet even begun vaccinating.

The latest episode of the vaccination saga came about at the end of January 2021, when the EU, as the first major trading power, introduced temporary export controls on COVID-19 vaccines covered by its Advance Purchase Agreements (APAs).^14^ Although Regulation 2021/111 is time limited (until 31 March 2021) and contains a broad exception clause (covering, for example, exports to low- and middle-income countries in the COVAX Advance Market Commitment (AMC) list), the decision has been widely criticised, particularly by those who fear that it will provoke other countries to take analogous steps. Indeed, since then, the press has reported that both the UK and the US governments are exploring similar options.^15^

One may ask: what is wrong with countries seeking ways to prioritise the health of their own citizens over foreigners? At the end of the day, these are the people to whom the respective governments are directly accountable. There are several reasons that speak against this approach, which is based on a narrow understanding of self-interests. The pragmatic reason, expressed by many public health experts, is connected with the mutation potential of SARS-CoV-2. Note that the risk of the emergence of new variants of the virus generally increases as the number of infections rises. Such variants may not only have higher transmissibility and mortality rates, but may also be more resistant to the currently available vaccines.^16^ As the examples of the South African (B.1.351) or Brazilian variants (P.1) show, sooner or later they will spread to other countries, potentially undermining the national vaccination efforts already undertaken. Vaccines should therefore be available in the first instance where the risk of exponential growth is the highest. Nor is a me-first approach advisable from the economic point of view. According to Rand Europe, its cost to the global economy may amount to $1.2 trillion a year in GDP terms.^17^ Considering how interconnected the world is, the impact will be felt by everyone, even by the developed countries with high vaccination rates. Last but not least, such a policy also raises important ethical questions. As was bitterly observed by the Director General of the World Health Organization (WHO): “The pandemic has exposed and exploited the inequalities of our world. There is now the real danger that the very tools that could help to end the pandemic – vaccines – may exacerbate those same inequalities.”^18^ Indeed, making vaccines available to individuals who are at low risk levels in the developed world ahead of high-risk groups in developing countries (eg people with underlying medical conditions or medical workers) is highly problematic,^19^ as is the use of wealth and power rather than needs as a primary allocation criterion.

Irrespective of how questionable from the ethical point of view vaccine nationalism is, it is doubtful whether, as a matter of principle, such conduct violates any rules of international law. Global health law, as it stands now, does not establish any specific obligations on States related to increasing the access of developing nations to health-related resources in situations when the needs of their domestic populations are not yet satisfied. In particular, there are no such rules in the WHO Constitution or International Health Regulations 2005, while international human rights treaties – as a part of their right to health – mainly concentrate on the obligations of a State vis-à-vis its own population.^20^ Even if such treaties impose some broader obligations (eg Articles 2(1) and 12 of the International Covenant on Economic Social and Cultural Rights), they are formulated in very general and ambiguous language that is subject to competing interpretations, with only a symbolic (if any) enforcement mechanism.^21^ Consequently, these treaties provide a weak basis for construing any enforceable right to equitable access by all peoples of all States to vaccines. This assessment is actually acknowledged in the recent statement of the Committee on Economic, Social and Cultural Rights, which recognises that “States give some priority to ensure access to vaccines for their own citizens first”.^22^ Although it also adds that “States must strengthen their international cooperation to guarantee, as soon as possible, universal and equitable access to vaccines for COVD-19 globally”^23^ and that “competition for vaccine is contrary to the extraterritorial obligations of States to avoid taking decisions that limit the opportunity of other states to implement their right to health”,^24^ it does not explain what is meant by “as soon as possible”, which type of conduct is actually prohibited, nor how to balance the needs of local and foreign populations.

Considering the realities of the current international legal system, it is not surprising that the various international actors, including the WHO, are trying to deal with the problem of vaccine nationalism through other (non-legal) means. We address this issue in the subsequent section.

## IV. Managing COVID-19 vaccine nationalism

In the early stage of the pandemic, some European countries (ie France, Germany, Italy and the Netherlands) formed the Inclusive Vaccine Alliance to accelerate the development of a COVID-19 vaccine. While the main objective of this initiative has been to secure access to a vaccine for European countries, it was also aimed at making a portion of vaccines available to low-income countries.^25^ More or less at the same time, another initiative – the so-called “Access to COVID-19 Tools (ACT) Accelerator” – has been launched by the WHO, the European Commission and France, which intends to bring together national governments, intergovernmental organisations, non-governmental organisations, civil society and private sectors to provide innovative and equitable access to COVID-19 diagnostics, treatments and vaccines. COVAX constitutes the vaccine pillar of the ACT Accelerator, aimed at ensuring equitable access to COVID-19 vaccines and prioritising high-risk and vulnerable people with the availability of 2 billion doses by the end of 2021.^26^

COVAX is co-led by the Vaccine Alliance (Gavi), WHO and the Coalition for Epidemic Preparedness Innovations (CEPI), and it is administered by Gavi. As explained by Dr Seth Berkeley, the CEO of Gavi, the objective of COVAX is to “maximise … chances of successfully developing COVID-19 vaccines and manufacture them in the quantities needed to end this crisis, and in doing so ensure that ability to pay does not become a barrier to accessing them”.^27^ To this end, COVAX secures the supply of vaccines through advanced market commitments by leveraging the amount committed by high-income countries, which also enables the participation of low-income states.^28^ Therefore, COVAX comprises two categories of participants: self-financing and funded. The first group includes high-income countries with or without the capacity to develop vaccines. Whereas some developed countries aim to develop their own vaccines and secure vaccine supplies through APAs with pharmaceutical companies, they may still wish to join COVAX as an insurance policy, as the initiative manages a wide portfolio of vaccine candidates across different technologies.^29^ Other developed countries that lack the capacity to develop their own vaccines join COVAX with a view to securing access to vaccines once they are on the market. Self-financing participants can join COVAX through two modes of arrangements: committed purchase arrangements and optional purchase arrangements.

By virtue of committed purchase arrangements, self-financing participants are asked to make an upfront payment of $1.6 per dose or $3.2 per person, as well as a financial guarantee of $8.95 per dose or $17.90 per person, and to procure the full amount of the doses of vaccines at the actual price with the manufacturers. Under these committed purchase agreements, self-financing countries are allowed to opt out only based on price. For this purpose, a participant has to indicate in its commitment agreement that it will not accept a price higher than double the all-inclusive estimate of $10.55 per dose.^30^ By contrast, under optional purchase arrangements, self-financing participants can decide whether to accept any approved vaccine allocated to them, but they are at the same time eligible for receiving other vaccines once they are available. Given the freedom to reject a vaccine allocated to it and the uncertainty arising therefrom, a self-financing participant under optional purchasing arrangements is required to make a higher upfront payment of $3.10 per dose or $6.20 per person. Moreover, it is asked to provide a risk-sharing guarantee of $0.40 per dose against the contractual liability arising from the decisions of self-financing participants opting not to accept the vaccine.^31^ As of 15 December 2020, sixty countries have signed committed agreements with the COVAX facility. The European Commission, acting on behalf of its twenty-seven Member States and Norway and Iceland, has also joined COVAX, together with eight “economies” that are not UN Members (including Taiwan).^32^ A notable initial absentee was the USA, which decided to stay away from the initiative.^33^ This changed, however, with the appointment of the Biden administration. The US reversed its earlier decision to withdraw from the WHO, and also joined COVAX.^34^

Funded participants, now comprising ninety-two low- and middle-income economies, are eligible for the COVAX facility supported by the COVAX AMC.^35^ The funding for vaccines for the funded participants comes from donor partners, philanthropy foundations and CEPI. With the inauguration of the Biden administration in 2021, leaders of the G7 countries, including the USA, pledged to strengthen their support for COVAX at the virtual meeting held on 15 February 2021.^36^ The first batch of vaccines under the COVAX AMC was delivered to Ghana on 24 February 2021.^37^

Based on its current performance, COVAX cannot thus far be adjudged to be a significant success. The aim behind the creation of COVAX has been to avoid panic buying out of self-interest (ie when high-income countries crowd out the needs of high-risk and vulnerable groups whether in high-income or low-income states). COVAX therefore represents a multilateral solution to the global pandemic with a view towards constraining self-interested unilateral acts.^38^ However, the hostility of the Trump administration towards the WHO (as reflected in its decision to abstain from the initiative), combined with the general process of the waning authority of the WHO in global health governance,^39^ did not encourage countries to rely on the allocation of vaccines through COVAX. But the USA is again not the only country to blame. While embracing the European values and endorsing multilateralism as a way of conducting international politics, the EU and some of its Member States (including Germany) have also joined the vaccine hoarding race and employed beggar-thy-neighbour policies. Although the EU has pledged to support COVAX and established its own vaccine-sharing mechanism, prioritising the Western Balkans, its eastern and southern neighbourhood as well as Africa,^40^ there seems to be quite a lot of hypocrisy in its actions, particularly if one recalls the export restrictions on COVID-19 vaccines and the EU’s reservation of a lion’s share of total vaccine production.

However, overbuying by high-income countries is not the only factor undermining the effectiveness of the COVAX initiative. The supply-side problem – namely, the production constraints of the various vaccine manufacturers – also accounts for the lack of significant progress in global vaccinations. To overcome this obstacle, close cooperation among pharmaceutical companies appears to be necessary. A good sign in this regard is the conclusion of two recent agreements between Novartis and Pfizer^41^ and between Sanofi and Johnson & Johnson, pursuant to which Novartis and Sanofi have agreed to the use of their manufacturing facilities to produce vaccines developed by Pfizer and Johnson & Johnson.^42^ Similarly, AstraZeneca has recently concluded a number of contract manufacturing agreements with drug manufacturing companies outside Europe to scale up the supply of the Oxford/AstraZeneca COVID-19 vaccine (AZD1222). In fact, the batch delivered to Ghana was made by the Serum Institute of India.^43^ Yet it remains to be seen whether these steps will significantly contribute to a more equitable access to the available vaccines.

## V. Conclusions

Vaccine nationalism has dominated the initial response to the pandemic in almost the entire developed world. Governments have mostly opted for short-term health, political and economic gains, privileging their own populations over those from developing countries. Various initiatives undertaken at the regional and global levels aimed at ensuring and facilitating a more equitable distribution of the vaccines have so far been rather unsuccessful. Although there are some signs that the situation may improve in the future, we are still far from any comprehensive solution.

Paradoxically, vaccine nationalism seems to be a phenomenon that is characteristic of the wealthy Western countries. The aspiring powers such as China and Russia (and, to a lesser extent, India) have decided to take a different path, seeing the vaccine crisis as an opportunity to advance their geopolitical goals rather than a misfortune. As a part of their vaccine diplomacy, these countries are willing to provide COVID-19 vaccines (ie Sputnik V, Sinopharm and Sinovac) to emerging or low-income countries in an attempt to cement the existing ties or to develop new alliances. This is done despite the fact that their respective domestic markets still remain undersupplied. Ironically, this could mean that the geopolitical rivalry is the best remedy for vaccine nationalism.

## Footnotes

1 Cf “Covid-19 Coronavirus Pandemic” (*Worldometer*) <https://www.worldometers.info/coronavirus/>. All electronic sources, unless specified otherwise, were last accessed on 11 March 2021.

2 See eg AFP, “Russia admits to world’s third-worst Covid-19 death toll” (*The Guardian*, 28 December 2020) <https://bit.ly/3rBTjpJ>; D Kobak, “Excess mortality reveals Covid’s true toll in Russia” (2021) 18 Significance 16.

3 T Adhanom Ghebreyesus, “Vaccine nationalism harms everyone and protects no one” (*Foreign Policy*, 2 February 2021) <https://bit.ly/3skqLAG>; DP Fidler, “Vaccine nationalism’s politics” (2020) 369 Science 749.

4 See eg H Kretchmer, “Vaccine nationalism – and how it could affect us all” (*World Economic Forum*, 6 January 2021) <https://www.weforum.org/agenda/2021/01/what-is-vaccine-nationalism-coronavirus-its-affects-covid-19-pandemic/>.

5 S Vella et al, “The history of antiretroviral therapy and of its implementation in resource-limited areas of the world” (2012) 26(10) AIDS 1231; see also R Love, “Corporate wealth or public health? WTO/TRIPS flexibilities and access to HIV/AIDS antiretroviral drugs by developing countries” (2007) 17(2) Development in Practice 208.

6 DP Fidler, “Negotiating equitable access to influenza vaccines: global health diplomacy and the controversies surrounding avian influenza H5N1 and pandemic influenza H1N1” (2010) 7(5) PLoS Medicine e1000247.

7 K Bennhold and DE Sanger, “U.S. offered ‘large sum’ to German company for access to coronavirus vaccine research, German officials say” (*The New York Times*, 15 March 2020) <https://nyti.ms/2MfKZwa>.

8 A Cozzolino, “Trumpism as nationalist neoliberalism. A critical enquiry into Donald Trump’s political economy” (2018) 4(1) Interdisciplinary Political Studies 47, 54.

9 MB Marcus, “Ensuring everyone in the world gets a COVID vaccine” (*Duke Global Health Institute*, 20 January 2021) <https://bit.ly/3pO1EVo>.

10 O Dyer, “Covid-19: Many poor countries will see almost no vaccine next year, aid groups warn” (2020) 371 BMJ m4809.

11 WTO, “Members to continue discussion on proposal for temporary IP waiver in response to COVID-19” (10 December 2020) <https://www.wto.org/english/news_e/news20_e/trip_10dec20_e.htm>.

12 WTO – Council for Trade-Related Aspects of Intellectual Property Rights, “Waiver from certain provisions of the TRIPS Agreement for the prevention, containment and treatment of Covid-19, Communication from India and South Africa” (2 October 2020), IP/C/W/669.

13 “Coronavirus (COVID-19) vaccinations” (*Our World in Data*, 9 March 2021) <https://ourworldindata.org/covid-vaccinations>. Note that when calculating the rates every single dose is counted. Consequently, the figures do not show the total number of people vaccinated, as some vaccines require two shots.

14 Commission Implementing Regulation (EU) 2021/111 of 29 January 2021 making the exportation of certain products subject to the production of an export authorization, OJ L 31I, 30.1.2021, pp 1–8. The first decision was taken on 4 March 2021 when Italy blocked the shipment of 250,000 AstraZeneca vaccines destined for Australia.

15 See eg K Mims, I Raphaelson and L Kim, “How defense production act may affect vaccine supply chain” (*Law 360*, 20 January 2021) <https://bit.ly/3qSakvd>.

16 Eg with respect to the South African variant, see SA Madhi et al, “Safety and efficacy of the ChAdOx1 nCoV-19 (AZD1222) Covid-19 vaccine against the B.1.351 variant in South Africa”, medRxiv 2021.02.10.21251247; doi: 10.1101/2021.02.10.21251247.

17 M Hafner et al, “COVID-19 and the cost of vaccine nationalism” (Santa Monica, CA, RAND Corporation 2020) <https://www.rand.org/pubs/research_reports/RRA769-1.html>.

18 WHO, “WHO Director-General’s opening remarks at the UNICEF Executive Board” (10 February 2021) <https://bit.ly/3qOIKij>.

19 See generally R Gupta and SR Morain, “Ethical allocation of future COVID-19 vaccine” (2021) 47 Journal of Medical Ethics 137.

20 DP Fidler, “International law and equitable access to vaccines and antivirals in the context of 2009-H1N1 influenza”, in DL Relman, EF Choffnes and A Mack (eds.), *The Domestic and International Impacts of the 2009-H1N1 Influenza A Pandemic: Global Challenges, Global Solutions* (Washington, DC, The National Academies Press 2010) p 143 et seq.

21 But see D Desierto, “Equitable COVID vaccine distribution and access: enforcing international legal obligations under economic, social, and cultural rights and the right to development” (*EJIL: Talk!*, 2 February 2021) <https://bit.ly/3qVWaZW>.

22 Committee on Economic, Social and Cultural Rights, “Statement on universal and equitable access to vaccines for COVID-19”, 27 November 2020, E/C.12/2020/2, para 10.

23
ibid, para 9.

24
ibid, para 10.

25 Government of the Netherlands, “France, Germany, Italy and the Netherlands working together to find a vaccine for countries in Europe and beyond” (3 June 2020) <https://bit.ly/3aZcZO5>.

26 COVAX, “COVAX: the vaccines pillar of the Access to COVID-19 Tools (ACT) accelerator structure and principles” (9 September 2020) <https://bit.ly/3q2wCck>, at 5.

27 S Berkeley, “COVAX explained” (*Gavi: The Vaccine Alliance*, undated) <https://bit.ly/3q0aPCi>.

28 S Berkeley, “The Gavi COVAX AMC explained” (*Gavi: The Vaccine Alliance*, undated) <https://bit.ly/3dTQD2A>.

29 WHO, “COVAX Facility Explainer: Participation Arrangements for Self-financing Economies” (undated) <https://bit.ly/2MwjUoB>, at 2.

30
ibid, at 4.

31
ibid, at 5.

32 WHO, “List of participating economies” (15 December 2020) <https://bit.ly/30aODel>, at 2–3.

33 A Rouw et al, “COVAX and the United States” (*KFF Issue Brief*, 18 February 2021) <https://bit.ly/3q5sMiK>.

34 S Nebehay, “U.S., staying in WHO, to join COVID vaccine push for poor nations: Fauci” (*Reuters*, 21 January 2021) <https://reut.rs/3q0I2xw>.

35 WHO, supra, note 32, at 6.

36 P LeBlanc, “Biden to join virtual G7 event Friday with global leaders on Covid-19 efforts” (*CNN*, 15 February 2021) <https://cnn.it/3dTPQyE>.

37 WHO, “COVID-19 vaccine doses shipped by the COVAX Facility head to Ghana, marking beginning of global rollout” (24 February 2021) <https://bit.ly/3bKKMd1>.

38 See eg A von Bogdandy and P Villarreal, “The EU’s and UK’s self-defeating vaccine nationalism” (*Verfassungblog*, 30 January 2021) <https://bit.ly/2OcFkYA>.

39 See also LO Gostin, S Moon and BM Meier, “Reimagining global health governance in the age of COVID-19” (2020) 110 American Journal of Public Health 1615.

40 Communication from the Commission to the European Parliament, the European Council and the Council: a united front to beat COVID-19, 19 January 2021, COM(2021) 35 final, p 10.

41 Novartis, “Novartis signs initial agreement to provide manufacturing capacity for Pfizer–BioNTech COVID-19 vaccine” (29 January 2021) <https://bit.ly/2ZZHMUE>.

42 Sanofi, “Sanofi to provide manufacturing support to Johnson & Johnson for their COVID-19 vaccine to help address global supply demands” (22 February 2021) <https://bit.ly/3bRJLAe>.

43 AstraZeneca, “Serum Institute of India obtains emergency use authorisation in India for AstraZeneca’s COVID-19 vaccine” (6 January 2021) <https://bit.ly/3b3mZ9k>.

